# Chirurg*innen vs. Wissenschaftler*innen – Mind the Gap! DKOU Science-Slam 2023: Umfragestudie zu Biomaterialien bei Knochendefekten

**DOI:** 10.1007/s00132-024-04492-9

**Published:** 2024-04-05

**Authors:** Tina Frankenbach, Susanne Mayer-Wagner, Wolfgang Böcker, Dietmar W. Hutmacher, Boris M. Holzapfel, Markus Laubach

**Affiliations:** 1 Klinik für Orthopädie und Unfallchirurgie, Muskuloskelettales Universitätszentrum München (MUM), LMU Klinikum, LMU München, München, Deutschland; 2 Max Planck Queensland Centre (MPQC) for the Materials Science of Extracellular Matrices, Queensland University of Technology, 4000 Brisbane, QLD Australien; 3 Australian Research Council (ARC) Training Centre for Multiscale 3D Imaging, Modelling, and Manufacturing (M3D Innovation), Queensland University of Technology, 4000 Brisbane, QLD Australien

## Infobox Wissenschaft trifft Humor

Das ist das Prinzip eines Science-Slams. Bei diesem Kurzvortragswettbewerb präsentieren Forschende ihre Themen auf unterhaltsame Weise innerhalb einer vorgegebenen Zeit dem Publikum. Dieses entscheidet, wem es am besten gelingt, Unterhaltung und Erkenntnisgewinn in Einklang zu bringen.

Der Science-Slam wird im Rahmen des DKOU jedes Jahr vom Jungen Forum O und U organisiert und veranstaltet. Das Siegerteam wird vom Publikum und einer wissenschaftlichen Jury nach den drei Kriterien Wissenschaftlichkeit, Spaßfaktor und Applauslautstärke gewählt. Die beste Darbietung wird mit dem Slam-Stipendium der DGOU in Höhe von 1000 € ausgezeichnet. Der Springer Medizin Verlag unterstützt den Science-Slam mit einem Jahresabo e.Med Orthopädie & Unfallchirurgie und drei Jahresabos e.Medpedia.

2023 ging der Science-Slam bereits in die 6. Runde, moderiert von Dr. Marie Samland. Alle vier Vorträge haben die „Slammer“ noch einmal in Form eines Beitrags für Sie aufbereitet.

Sie möchten sich selbst ein Bild von dem Format machen? Schauen Sie sich gerne den Science-Slam-Rückblick vom DKOU 23 an, Link: https://dgou.de/aktuelles/detail/science-slam-dr-thomas-loy-ueberzeugt-mit-gschichten-vom-venusberg.


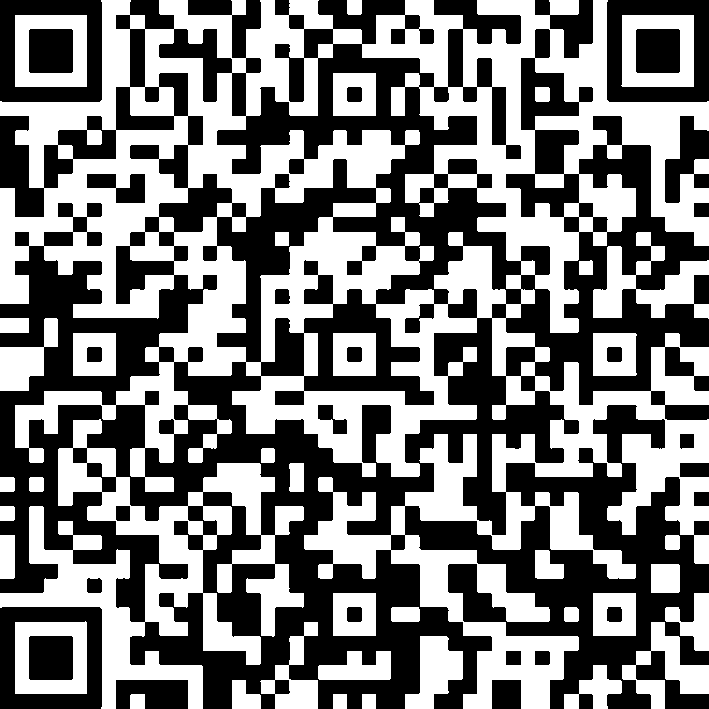


Für wirksame und kosteneffiziente Knochendefektbehandlung ist es von Bedeutung, die Entscheidungsprozesse von Chirurg*innen und Wissenschaftler*innen in Bezug auf aktuelle Optionen und künftige Möglichkeiten der Biomaterial- und Implantatentwicklung zu verstehen. Im Rahmen des DKOU 2023 Science-Slam wurden, insbesondere für Strategieentscheidungen der Medizintechnologieindustrie und Forschungsinstitute, wegweisende Ergebnisse einer Umfragestudie und konkrete Konzeptempfehlungen vorgestellt.

## Mind the Gap! Translationslücke im Bereich Biomaterialien und Implantate für Knochendefekte

**Figure 1:**
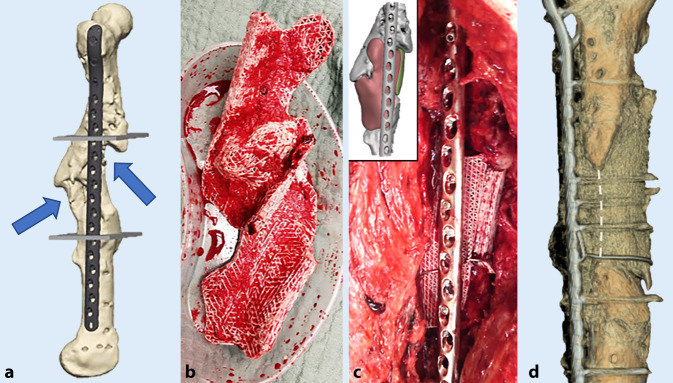

Die Behandlung von Knochendefekten ist nach wie vor eine klinische Herausforderung, die mit hohen Reinterventionsraten, einer hohen Patientenmorbidität und erheblichen Kosten im Gesundheitswesen assoziiert ist [1]. Sowohl chirurgische Techniken als auch nichtstrukturelle und strukturelle Knochenersatzmaterialien werden ständig weiterentwickelt; so konnten etwa kürzlich erste klinische Erfahrungen mit biologisch abbaubaren 3D-gedruckten Knochengerüsten (sog. Knochen-Scaffolds) der Literatur entnommen werden [2–4]. Seit dem oft als bahnbrechend bezeichneten Workshop für die Biomaterialienwissenschaft im Jahr 1987 in Washington, D.C., USA, der von der National Science Foundation gesponsert wurde und bei dem der Begriff Tissue Engineering zum ersten Mal offiziell geprägt wurde [5], wurden beispielsweise mehr als 50.000 Studien zum Thema Scaffolds veröffentlicht, von denen sich mehr als 40 % auf das Tissue Engineering von Knochen beziehen [6]. Das Konzept des Knochen-Tissue-Engineering wurde erstmals 1993 von Langer und Vacanti vorgestellt und in einem wegweisenden Manuskript in *Science* veröffentlicht [7]. So bietet das patientenspezifische gerüstträgerbasierte Knochen-Tissue-Engineering („scaffold-guided bone regeneration“ [SGBR]) die Möglichkeit der Regeneration knöcherner Defekte [8]. Biodegradierbare bzw. bioresorbierbare Knochen-Scaffolds gewährleisten so lange strukturelle und biomechanische Stabilität, bis der Degradations- bzw. Resorptionsprozess einsetzt und körpereigenes Gewebe deren Funktion schrittweise übernimmt. Knochen-Scaffolds werden durch Verfahren der additiven Fertigung 3D-gedruckt, was die patientenspezifische Konfiguration der Implantatarchitektur ermöglicht [9]. So können die notwendige Gewebe(neu)formation und -regeneration über einen Zeitraum bis zu einigen Jahren kontrolliert werden, während simultan die entsprechende Knochenregeneration abläuft (Abb. 1; [10]).

**Figure 2:**
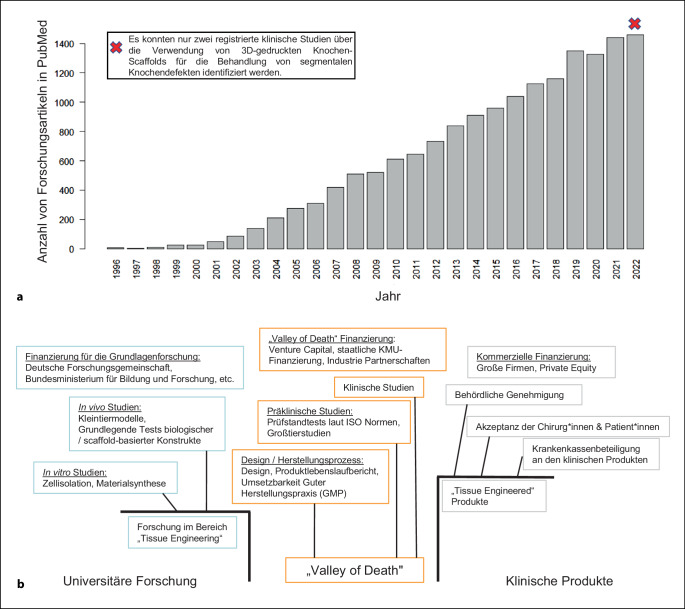

Jedoch werden trotz intensiver Forschung – im Jahr 2023 wurden immerhin mehr als 20.000 Studien zum Thema Knochen-Scaffolds und Knochen-Tissue-Engineering veröffentlicht – nur eine Handvoll Innovationen insofern in die klinische Praxis umgesetzt, dass diese tatsächlich „from bench to bedside“ gelangen [11, 12]. Somit ist eine persistierende und kostspielige Translationslücke („translational gap“) zu beobachten ([13, 14]; Abb. 2). Daher war es Ziel dieser dem Science-Slam-Beitrag zugrundeliegende Forschungsarbeit, diese Translationslücke näher zu untersuchen, um substanzielle Rahmenbedingungen für zielgerichtete und (kosten)effiziente zukünftige Forschungsanstrengungen im Bereich Knochenersatzmaterialien aufzuzeigen.

## Chirurg*innen vs. Wissenschaftler*innen – sprechen wir überhaupt dieselbe Sprache?

Chirurg*innen und Wissenschaftler*innen verfügen, basierend auf ihrer akademischen und praktischen Ausbildung, über Expertise in höchst unterschiedlichen Gebieten. Der Arbeitsalltag beider Berufsgruppen gestaltet sich gleichermaßen spezifisch, sie interagieren regelmäßig mit Arbeitsgruppen mit unterschiedlicher Expertise und kommerziellen Interessen aus dem Gesundheitssektor und haben möglicherweise sogar unterschiedliche konzeptionelle und wissenschaftliche Sichtweisen [15]. Diese Herausforderungen sind insbesondere etwa in der Mitte des letzten Jahrhunderts entstanden, denn bis etwa zu den 1950er- und 1960er-Jahren waren Grundlagenforschung und klinische Forschung in Einrichtungen sehr eng miteinander verbunden [16]. So wurde etwa die medizinische Forschung weitgehend von ärztlichen Wissenschaftler*innen betrieben, die auch Patient*innen behandelten. Das änderte sich mit der rasanten Zunahme und Spezialisierung, insbesondere im Bereich der Molekularbiologie in den 1970er-Jahren. Die klinische Forschung und die Grundlagenforschung begannen sich zu trennen, und die biomedizinische Forschung entwickelte sich zu einer eigenständigen Disziplin mit einer eigenen Ausbildung. So wird der größte Teil der biomedizinischen Forschung heute von hochspezialisierten (promovierten) Wissenschaftler*innen durchgeführt, während Mediziner*innen in diesen Forschungsgruppen in der Minderheit sind [16]. Ein weiterer wichtiger Faktor wurde erstmals von C. P. Snow in seiner Rede-Vorlesung (Rede Lecture) an der Universität Cambridge im Jahr 1959 thematisiert: In seinem berühmten Aufsatz „The Two Cultures“ bezeichnet er die Kommunikationsbarriere und das gegenseitige Unverständnis zwischen verschiedenen akademischen Disziplinen als Haupthindernisse für kollektives intellektuelles Wachstum und die Anwendung von umfassendem Wissen auf komplexe Probleme der realen Welt [17, 18].

Diese Bewertungsdifferenzen zwischen Chirurg*innen und Wissenschaftler*innen können eine mögliche Erklärung dafür sein, dass so wenige Innovationen tatsächlich „from bench to bedside“ gelangen. Dementsprechend wurde die Hypothese aufgestellt, dass diese Translationslücke auf die Tatsache zurückzuführen ist, dass Chirurg*innen und Wissenschaftler*innen sehr unterschiedliche Präferenzen und Sichtweisen in Bezug auf den Stand der Technik und das Innovationspotenzial von Behandlungsmöglichkeiten, insbesondere von (3D-gedruckten) Biomaterialien für Knochendefekte, haben. Um unsere Hypothese zu prüfen, haben wir eine webseitenbasierte Umfragestudie durchgeführt.

## Ergebnisse der webseitenbasierten Umfragestudie unter Chirurg*innen und Wissenschaftler*innen

**Figure 3:**
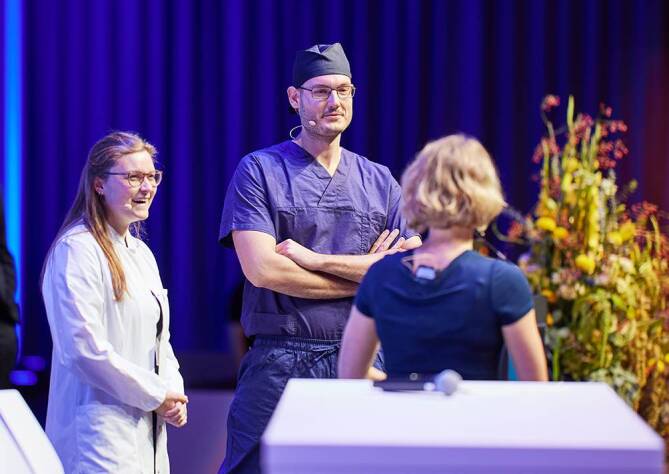

Es erfolgte eine webseitenbasierte Querschnittserhebung mittels elektronischer Fragebögen. Chirurg*innen und Wissenschaftler*innen wurden über Netzwerkkontakte und Konferenztreffen rekrutiert und eingeladen, eine Online-Umfrage zum Thema Entscheidungsfindung bei Knochendefektbehandlung („Decision making in bone defect treatment – What is your opinion?“) auszufüllen. Dabei wurde untersucht, inwieweit sich die Präferenzen und Perspektiven in Bezug auf die Möglichkeiten und die Entwicklung von autologen Knochentransplantaten und Knochenersatzprodukten, die regulatorischen und medikolegalen Herausforderungen von 3D-gedruckten Knochen-Scaffolds und die Hindernisse für die Einführung von 3D-gedruckten Medizinprodukten zur Behandlung von Knochendefekten zwischen diesen beiden Gruppen unterscheiden. Alle Daten wurden im Zeitraum vom 22.10.2022 bis 13.03.2023 gesammelt und unter anderem auf dem Deutschen Kongress für Orthopädie und Unfallchirurgie (DKOU) 2023 im Rahmen des Science-Slam vorgestellt (Abb. 3).

Wie in unserem Science-Slam-Beitrag herausgearbeitet, konnten wir beobachten, dass obwohl die Wissenschaftler*innen im Vergleich zu den Chirurg*innen deutlich jünger waren, beide Gruppen über umfangreiche Erfahrungen mit der Behandlung von Knochendefekten und im Umgang mit Biomaterialien, inklusive solcher aus dem Bereich des Knochen-Tissue-Engineering, hatten. Die Ergebnisse der Umfragestudie haben aufgezeigt, dass insbesondere Wissenschaftler*innen seltener an interdisziplinären Treffen teilnehmen, in deren Rahmen Patientenkasuistiken mit komplexen Knochendefekten besprochen werden. Wissenschaftler*innen waren häufiger der Meinung, dass autologe Knochentransplantate in Zukunft durch Knochenersatzprodukte ersetzt werden können und die entsprechende Knochenersatzproduktentwicklung vielversprechend sei. Sowohl Chirurg*innen als auch Wissenschaftler*innen schätzen die Notwendigkeit von vermehrten präklinischen (Groß)Tierstudien, klinischen Studien als auch der medikolegalen Rechtssicherheit als sehr groß ein, bevor 3D-gedruckte Scaffolds zur Knochenregeneration regelhaft eingesetzt werden können.

## Diskussion und Ausblick

**Figure 4:**
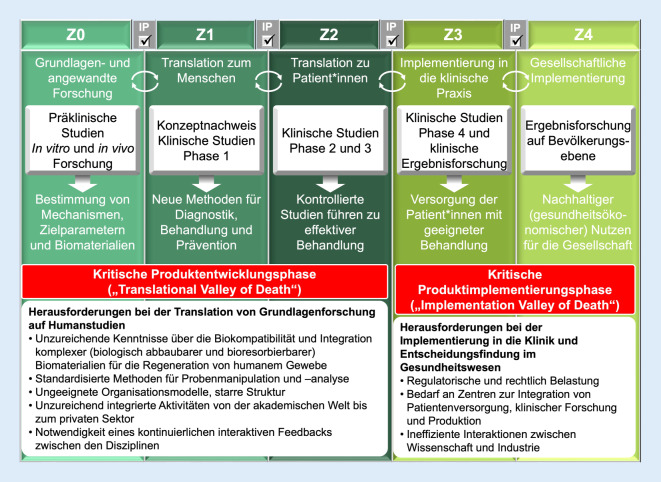

Die wertvollen Eigenschaften wie Automatisierung, Geschwindigkeit, Reproduzierbarkeit, Flexibilität bei kleinen Chargen und (potenziell) niedrige Herstellungskosten machen prinzipiell die jüngsten Entwicklungen im Bereich Knochenersatzprodukte und im 3D Druck für orthopädisch-unfallchirurgische Anwendungen sehr attraktiv und schaffen damit die Grundlage für die erfolgreiche Umsetzung vom Labor in die klinische Praxis. Bemerkenswerterweise konnten relevante unternehmerische Herausforderungen für die erfolgreiche Translation in die klinische Anwendung von Knochenersatzprodukten und 3D-gedruckten Knochen-Scaffolds, die noch vor etwa 15 Jahren im „translationalen Tal des Todes“ („Translational Valley of Death“) ([11]; Abb. 2 und 4) beschrieben wurden, inzwischen erfolgreich adressiert werden: (1) die behördliche Zulassung in vielen Ländern konnte verhandelt und patientenorientierte Lösungen gefunden werden und (2) sinnvoll eingesetzte Drittmittelallokationen mit der Finanzierung von (konfirmatorischen) Großtierstudien [19, 20] sowie klinischen Studien [21] können zunehmend beobachtet werden. Nichtsdestotrotz klafft weiterhin zwischen den unzähligen präklinischen Studien und der Anzahl an klinisch tatsächlich verwendeten Knochenersatzprodukten und 3D-gedruckten Knochen-Scaffolds für die Behandlung von Knochendefekten noch eine große Lücke [22]. Diese Translationslücke ist insbesondere relevant vor dem Hintergrund, wie langwierig und kostspielig der Produktentwicklungsprozess ist (Tab. 1). Selbst wenn der steinige Instanzenweg von Forschung, Entwicklung und Zulassung erfolgreich durchlaufen wird, ist das noch keine Garantie dafür, dass ein Produkt klinisch erfolgreich ist oder es überhaupt in die routinemäßige klinische Anwendung schafft, denn Einflussfaktoren wie die Entscheidungsprozesse von Chirurg*innen und Wissenschaftler*innen müssen erst im Detail verstanden werden, um auch die „kritische Produktimplementierungsphase“ („Implementation Valley of Death“) erfolgreich zu gestalten (Abb. 4; [22]).

**Table 1:** 

FDA-Zulassung: Produkte			
	Zulassung vor dem Inverkehrbringen (IDE/PMA)		FDA – 510(k)
	Präklinische Studien	Klinische Studien	Präklinische Studien
Zeit (in Jahren)	3–4	2–4	3–6
Kosten (in Millionen US-Dollar)	5–50	40–100	1–50
Ziel	Konzeptentwicklung, *in vitro* und *in vivo* Tests zur Bestimmung der Wirksamkeit und Sicherheit sowie zum Nachweis des Produktkonzeptes	Sicherheit und Wirksamkeit des Produkts, Bedingungen für die Verwendung des Produkts und seine Zuverlässigkeit	Nachweis der weitgehenden Gleichwertigkeit mit vorherigen 510(k) Produkten

*FDA* United States Food and Drug Administration

Unsere prospektive Umfrage zur Einschätzung der Entwicklung künftiger Biomaterialien und (3D-gedruckter) Implantate für Knochendefekte war daher ein passendes Instrument, um relevante Informationen hinsichtlich des Status quo, für die strategische Vorausschau und die politische Entscheidungsfindung zu sammeln und mehr über künftige Entwicklungen, Meinungen und Verhaltensweisen von Chirurg*innen und Wissenschaftler*innen zu erfahren. Künftige Strategien und Produkte zur Knochenregeneration sollten nachweislich folgendes bieten (1) eine überlegene Knochenregeneration mit ausgezeichneten klinischen Langzeitergebnissen und (2) vergleichbare Kosten zu bestehenden Methoden [23]. Daher ist das Verständnis der Entscheidungsprozesse von Chirurg*innen und Wissenschaftler*innen in Bezug auf aktuelle Optionen und künftige Möglichkeiten zur Behandlung von Knochendefekten von entscheidender Bedeutung für eine (kosten-)effektive Produktentwicklung.

**Figure 5:**
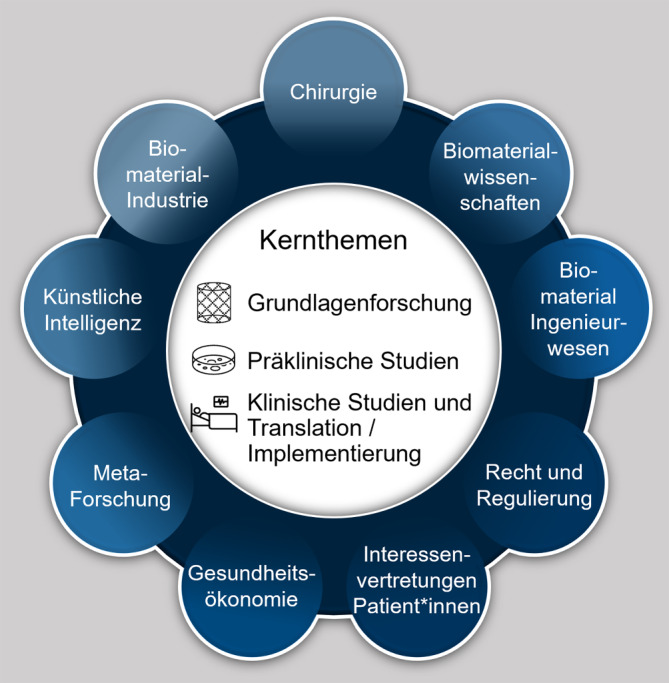

Um zukünftige Fehlallokationen von Arbeitsleistungen und Finanzmitteln zu vermeiden, sollten die aufgezeigten Diskrepanzen zwischen Chirurg*innen und Wissenschaftler*innen in Bezug auf die klinische Umsetzung von Biomaterialien und Implantate für Knochendefekte wie folgt angegangen und potenzielle Möglichkeiten zwischen den verschiedenen Fachbereichen genutzt werden:

- Da der globale Markt für Knochentransplantate und -ersatzprodukte signifikant wachsen wird, sind verstärkte Interaktionen und regelmäßige interdisziplinäre Treffen zwischen Chirurg*innen und Wissenschaftler*innen mit Vertreter*innen der Medizinprodukteindustrie eine Notwendigkeit. Das Ziel sollte sein, Fehlinvestitionen von (Forschungs‑)Mitteln in Knochendefektprojekte ohne praktische Relevanz zu vermeiden und Projekte stärker auf die klinische und wirtschaftliche Umsetzbarkeit auszurichten. Es gilt also die Zusammenarbeit fachdisziplinübergreifend, insbesondere zwischen Chirurg*innen und Wissenschaftler*innen, zu intensivieren. Dieses Credo ist nicht neu und wird seit mindestens 75 Jahren von einzelnen Schlüsselfiguren gefordert [24]. Eine fachdisziplinübergreifende Zusammenarbeit bedeutet nicht nur einen regelmäßigen Austausch, sondern das Eintauchen in den Alltag der jeweils anderen Disziplin, um die „Sprache“ zu erlernen, die entscheidend ist, um eine Basis für einen hochwertigen interdisziplinären Austausch zu schaffen [24]. Dazu ist es nicht zwingend erforderlich, in der jeweils anderen Disziplin ein „Meister“ zu werden, wie schon Goethe feststellte: „Wer mit unzulänglichem Talent sich der Musik bemüht, wird freilich nie ein Meister werden, aber er wird dabei lernen, dasjenige zu erkennen und zu schätzen, was der Meister gemacht hat.“ [25]. Im Rahmen dessen ist es möglicherweise auch eine Option, wissenschaftlich tätige Chirurg*innen und insbesondere Wissenschaftler*innen nicht nur nach ihren Publikationen, sondern auch nach betriebswirtschaftlichen Kriterien wie Meilensteinen und der Fähigkeit, in multidisziplinären Gruppen zu arbeiten, zu beurteilen [16]. Um entsprechend bereits jetzt in der Produktentwicklungs- und Implementierungsphase zu unterstützen, könnte es hilfreich sein, konkrete Ablaufschemata zur Anwendung zu bringen, um wichtige Meilensteine insbesondere durch interdisziplinären Austausch zu fazilitieren (Abb. 4).
- Eine interdisziplinäre Konsenssitzung ist dringend erforderlich, um prospektive Untersuchungen von Knochenersatzprodukten und (3D-gedruckten) Implantaten wie zum Beispiel Knochen-Scaffolds für die Behandlung von Knochendefekten anzugehen und spezifische chirurgische Indikationen, durchzuführende (prä-)klinische Studien, relevante medikolegale Aspekte und Lösungen für die Kostenerstattung zu diskutieren. Wissenschaft und Innovation sind zu komplex geworden, als dass Chirurg*innen und Wissenschaftler*innen ohne die notwendigen Synergien von fachdisziplinübergreifenden Kooperationen erfolgreich die Translation und Implementierung von Produkten in die klinische Praxis vorantreiben können [16]. Diese Kultur der fachdisziplinübergreifenden Zusammenarbeit zu etablieren und intensivieren ist substanziell, um Forschungsanstrengungen für die nächsten Jahrzehnte zu gestalten. Deshalb haben wir mit unserer Forschungsgruppe eine mehrtägige multidisziplinäre Konsenssitzung für Februar 2024 terminiert (Abb. 5).

## Fazit für die Praxis

- Wir haben eine webseitenbasierte Umfragestudie durchgeführt, die signifikante Diskrepanzen zwischen Chirurg*innen und Wissenschaftler*innen in der Einschätzung von für die Knochendefektbehandlung relevanten Therapien mittels autologen Knochentransplantaten, Knochenersatzprodukten und 3D-gedruckten Implantaten aufzeigte.
- Basierend auf den Ergebnissen, scheint eine Fehlallokationen von Arbeitsleistung und Finanzmitteln in der Medizintechnologieindustrie und in Forschungsinstituten im Bereich Biomaterialien und 3D-gedruckter Implantate für Knochendefekte wahrscheinlich.
- Um die erfolgreiche Erforschung und Translation von Innovationen auf dem Gebiet der Biomaterialien und 3D-gedruckten Implantate für Knochendefekte unmittelbar zu unterstützen, wurde in unserer translationalen Forschungsgruppe ein Ablaufschema zur iterativen Evaluation der notwendigen Interdisziplinarität und ein Konzept für eine interdisziplinäre Konsenssitzung erstellt.
